# Trauma care inside and outside business hours: comparison of process quality and outcome indicators in a German level-1 trauma center

**DOI:** 10.1186/s13049-014-0062-2

**Published:** 2014-10-31

**Authors:** Wolfgang Parsch, Markus Loibl, Uli Schmucker, Franz Hilber, Michael Nerlich, Antonio Ernstberger

**Affiliations:** Department of Trauma Surgery, University Hospital Regensburg, Franz-Josef-Strauß-Allee 11, 93042 Regensburg, Germany

**Keywords:** Multiple trauma patient, Office/business hours, Out of hours, Process quality management, Severely injured patient

## Abstract

**Background:**

Optimal care of multiple trauma patients has to be at a high level around the clock. Trauma care algorithms and guidelines are available, yet it remains unclear if the time of admission to the trauma room affects the quality of care and outcomes. Hence the present study intends to compare the quality of trauma room care of multiple severely injured patients at a level-1 trauma center depending on the time of admission.

**Methods:**

A total of 394 multiple trauma patients with an ISS ≥ 16 were included into this study (observation period: 52 months). Patients were grouped by the time and date of their admission to the trauma room [business hours (BH): weekdays from 8:00 a.m. to 4:00 p.m. vs. non-business hours (NBH): outside BH]. The study analysed differences in patient demographics, trauma room treatment and outcome.

**Results:**

The study sample was comparable in all basic characteristics [mean ISS: 32.3 ± 14.3 (BH) vs. 32.6 ± 14.4 (NBH), p = 0.853; mean age: 40.8 ± 21.0 (BH) vs. 37.7 ± 20.2 years (NBH), p = 0.278]. Similar values were found for the time needed for single interventions, like arterial access [4.8 ± 3.9 min (BH) vs. 4.9 ± 3.4 min (NBH), p = 0.496] and quality-assessment parameters, like time until CT [28.5 ± 18.7 min (BH), vs. 27.3 ± 9.5) min (NBH), p = 0.637]. There was no difference for the 24 h mortality and overall hospital mortality in BH and NBH, with 13.5% vs. 9.1% (p = 0.206) and, 21.9% vs. 15.4% (p = 0.144), respectively. The Glasgow Outcome Scale (GOS) comparison revealed no difference [3.7 ± 1.6 (BH) vs. 3.9 ± 1.5 (NBH), p = 0.305]. In general, the observed demographic, injury severity, care quality and outcome parameters revealed no significant difference between the two time periods BH and NBH.

**Conclusions:**

The study hospital provides multiple trauma patient care at comparable quality irrespective of time of admission to the trauma room. These results might be attributable to the standardization of the treatment process using established principles, algorithms and guidelines as well as to the resources available in a level-1 trauma center.

## Background

Trauma is one of the most frequent causes of death and disability around the world [1]. According to the Global Burden of Disease study 10% of all deaths result from injury [2]. Therefore, many trauma education initiatives have been implemented aiming at reducing mortality. Especially when the multiple severely injured is concerned, trauma care quality has to be maintained efficiently on a constant high level around the clock [3].

The “golden hour of shock” - that was postulated already in 1976 - emphasizes the significance of time and time loss [4]. Consequently a set of quality management related structures and processes were developed and in the past four decades:

### Trauma care algorithms and training

Studies consistently report that a trauma room algorithm has a positive effect on patient care [5-7]. Concepts like Advanced Trauma Life Support (ATLS) or European Trauma Course (ETC) aim at ensuring efficient trauma room care workflows and preventing secondary injury [8].

The regular trauma room treatment of the study hospital is based on the ATLS-algorithm and is divided into three parts:

1. Resuscitation phase with first and second survey
2. Whole-body Computed Tomography (CT)
3. Finalization of diagnostics and determination of further treatment

### Trauma registries for quality management and research

Trauma registries have been established as a tool for quality assessment over the last decades. In Germany, the TraumaRegister DGU® (TR-DGU) of the German Trauma Society (DGU) has been invented more than 20 years ago in order to collect and process data on the prehospital and clinical course. Each of the 600+ participating hospitals receive an annual report [3]. Comparable trauma registers are available, i.e. the Trauma Audit & Research Network (TARN) in the UK [9].

### Establishment of designated trauma centers

Usually hospitals are categorized as either Level-1, -2 or -3 trauma center. There is evidence from previous studies that a trauma system with designated trauma centers improves the survival rate and reduces the mortality rate by up to 15% [10-12].

The requirements for a level-1 trauma center are: [13]

1. 24/7 emergency department and trauma room service
2. 24/7 intensive care service including 24/7 admission of referred patients
3. 24/7 emergency surgery service
4. Immediate 24/7 availability of all specialists needed for interdisciplinary trauma care

Nevertheless, patient care including trauma care is usually forced into a system of business (BH) and non-business hours (NBH).

It is common sense that the overall resources available for trauma care (i.e. manpower) are substantially higher during BH compared to NBH, when many trauma team members are on duty.

The aim of this study was to evaluate the quality of multiple severely injured trauma care in BH and NBH. Differences in patient demographics, quality indicators in trauma room treatment (e.g. time to CT), and outcome parameters were assessed. To the authors’ knowledge, this is the first study that evaluates the quality of trauma care at various hours, which includes a set of time periods to complete a certain intervention from a large study sample.

## Methods

### Study Sample

The inclusion criteria are listed below:

- Admission to trauma room during the study period (September 2007 - December 2011)
- Presence of a trained research follower for case documentation
- Injury Severity Score (ISS) ≥ 16

A total of 757 patients were admitted to our trauma room during the study period, of which 499 presented with an ISS ≥ 16. A research fellow was not present in 105 of these 499 cases in the trauma room the entire time for various reasons; therefore these data sets were not used in this study. The remaining 394 cases are henceforth referred to as “study sample”.

The study sample was divided into the BH sample (weekdays [8:00 am; 4:00 pm]) and the NBH sample (weekdays]4:00 pm; 8:00 am[or weekends or public holidays).

The catchment area of the study hospital (university hospital and level-1 trauma center) covers approximately 20.000 km² with a population of two million residents. Ground based and helicopter air rescue services are available on a 24/7 basis. The helicopter is used for air rescue and intensive care patient transport. During the night, air rescue can only be called by the emergency physician on scene [14].

### Trauma team and care algorithm

The trauma room team comprises one trauma surgery consultant and one resident, one radiologist with one radiographer, one anesthesiologist with one nurse and two emergency nurses. Other disciplines (i.e. neurosurgery) can be consulted at any time. During NBH, these consultants are on call.

The intra-hospital workflow begins with the announcement of the trauma patient by the rescue coordination center. Such announcements trigger a set of pre-determined workflows, including the immediate call of consultants required for the case. Upon arrival of the patient, the history and clinical course are presented by the emergency physician. Thereafter, the primary survey begins. The aim of this primary survey is to rapidly resuscitate and examine the patient according to the ABCDE-concept [15]. The secondary survey starts after the stabilization of all vital functions and aims at identifying all anatomic injuries [16]. If stable, the patient is then transferred to the adjacent CT room and a whole-body CT is performed. Upon completion, the patient is transported back to the trauma room for a second trauma room phase.

### Data acquisition

The whole trauma room phase was documented by a trained research fellow, who was also in charge of capturing the pre-hospital data from the respective patient records.

A total of 450 items were recorded in each patient, of which 130 were the standard data set of the trauma registry of the German Trauma Society (TraumaRegister DGU®). The fundamental part was the 130 items of the TR-DGU. The data inputted into the TR-DGU contained information about patient demographics and defined treatment time points: pre-hospital, trauma room, ICU and discharge. Furthermore, the TR-DGU documents the diagnosis and grade of injury according to the Abbreviated Injury Scale (AIS) [17], Injury Severity Score (ISS) [18], New Injury Severity Score (NISS) [19], Trauma and Injury Severity Score (TRISS) [20] and the Revised Injury Severity Classification Score (RISC-Score) [21]. Moreover, the ASA physical status classification system is recorded for each patient [22]. Furthermore, the time points for interventions and operations, and outcome according to the Glasgow Outcome Scale (GOS) [23]) were gathered [24].

The additional 320 items precisely recorded the time to and the duration of a broad spectrum of diagnostic and therapeutic interventions (e.g. chest tube, intubation, intravenous lines or splinting of the extremities) until admission to the ICU, transport to the operating room, or death.

In addition, all emergency physicians were asked to complete a standardized questionnaire on the perceived quality of the trauma room team on charge and available equipment.

### Statistics

Statistical analysis was performed with IBM SPSS 21.0.0 for Windows. Normality was tested with the Kolmogorov-Smirnov test. The Chi-square test was used to analyze dichotomous variables, the unpaired *t*-test and Mann–Whitney *U* test were used to analyze continuous variables. Data are presented as mean ± standard deviation. p-values < 0.05 were considered statistical significant in all tests. An outcome analysis was performed by calculating the Standardized Mortality Ratio (SMR) of the BH and NBH patients for their TRISS- and RISC-prognosis.

### Ethical considerations

The study was approved by the Institutional Review Board of the University of Regensburg (Number 14-101-0004).

## Results

### Demography

The study sample consists of 394 patients. 96 (24%) of these were admitted to the hospital during BH and 298 (76%) patients were admitted during NBH. Significantly more males than females (72% versus 28%) were included. The mean age was 38.5 + 20.4 years (Table 1).

**Table 1:** **Basic characteristics: Patients assigned to groups depending on their time and date of arrival in the trauma room**

	**n (total)**	**mean (total) ± SD (total)**	**n (BH)**	**mean (BH) ± SD (BH)**	**n (NBH)**	**mean (NBH) ± SD (NBH)**	**p-value**
Age (years)	394	38.5 ± 20.4	96	40.8 ± 21.0	298	37.7 ± 20.2	0.278
Male (%)	285 (72.3%)		62 (64.6%)		223 (74.8%)		0.510
ISS	394	32.5 ± 14.3	96	32.3 ± 14.3	298	32.6 ± 14.4	0.853
NISS	394	39.5 ± 16.1	96	40.2 ± 16.5	298	39.3 ± 16.0	0.675
TRISS	302	0.72 ± 0.34	75	0.71 ± 0.36	227	0.72 ± 0.33	0.923
RISC	388	24.1 ± 30.9	95	28.4 ± 33.5	293	22.7 ± 29.9	0.327
ASA	248	1.3 ± 0.6	54	1.3 ± 0.6	194	1.4 ± 0.6	0.251

ASA: American Society of Anesthesiologists Classification, ISS: Injury Severity Score, NISS: New Injury Severity Score, RISC: Revised Injury Severity Classification Score, TRISS: Trauma and Injury Severity Score.

### Injury severity

The mean ISS was 32.5 ± 14.3 [BH: 32.3 ± 14.3, NBH: 32.6 ± 14.4, p = 0.853] with no significant difference between BH and NBH. Comparably, there was also no significant difference for the NISS, TRISS, RISC or ASA Classification, p ≥ 0.251 (Table 1).

Similarly, there was no difference between BH and NBH for the patients who received a whole-body CT or were treated with a mass-transfusion (> 10 erythrocyte concentrate (EC)) (Table 1).

Regarding the type of transport, helicopter transport was significantly more frequent in the BH sample compared to the NBH sample [BH: n = 65 (68% of all 96 BH patients), NBH: n = 167 (56% of all 298 NBH patients), p = 0.025]. Table 2 demonstrates that other prehospital parameters do not show any statistical difference.

**Table 2:** **Comparison of prehospital parameters of BH and NBH**

	**n (Total)**	**mean (total) ± SD (total)**	**n (BH)**	**mean (BH) ± SD (BH)**	**n (NBH)**	**mean (NBH) ± SD (NBH)**	**p -value**
Air-rescue (%)	232		65		167		0.025
	(58.8%)		(67.7%)		(56.0%)		
Prehospital intubation (%)	286		67		219		0.450
	(72.6%)		(69.8%)		(73.5%)		
Initial GCS	323	10.0 ± 4.7	80	10.0 ± 4.7	243	10.0 ± 4.7	0.964
Heart rate prehospital (1/min)	315	94.1 ± 30.6	79	92.3 ± 35.8	236	94.7 ± 28.7	0.587
RR (sys) prehospital (mmHg)	311	108.9 ± 39.6	77	105.6 ± 42.9	234	110.0 ± 38.5	0.419
RR ≤ 90 mmHg prehospital (%)	98		29		69		0.180
	(31.5%)		(37.7%)		(29.5%)		
SpO_2_ prehospital (%)	290	90.4 ± 15.6	68	88.6 ± 19.9	222	91.0 ± 14.0	0.693

The proportion of patients with prehospital RR ≤ 90 mmHg is calculated from the patients with available blood pressure values. RR: Blood pressure.

The parameters recorded at the admission of the patients didn’t differ in any aspect. Overall, 62 patients (16.0%) were in shock (systolic blood pressure ≤ 90 mmHg) on hospital admission [BH: n = 20 (21.1%), NBH: n = 42 (14.4%), p = 0.124] (Table 3).

**Table 3:** **Comparison of parameters on arrival of BH and NBH**

	**n (Total)**	**mean (total) ± SD (total)**	**n (BH)**	**mean (BH) ± SD (BH)**	**n (NBH)**	**mean (NBH) ± SD (NBH)**	**p -value**
Time accident to admission (min)	328	89.8 ± 37.1	81	83.6 ± 26.3	247	91.8 ± 39.9	0.367
Body temperature on arrival (°C)	333	35.9 ± 1.3	82	35.8 ± 1.2	251	35.9 ± 1.3	0.149
Heart rate on arrival (1/min)	388	89.9 ± 23.0	95	90.1 ± 22.5	293	89.9 ± 23.2	0.952
RR systolic on arrival (mmHg)	387	118.9 ± 32.1	95	116.5 ± 32.2	292	119.7 ± 32.0	0.391
RR ≤ 90 mmHg on arrival (%)	62 (16.0%)		20 (21.1%)		42 (14.4%)		0.124
SpO_2_ on arrival (%)	380	96.7 ± 9.0	93	97.0 ± 5.5	287	96.6 ± 9.9	0.557
Transfusion ≥ 10 ECs (%)	17 (4.3%)		7 (7.3%)		10 (3.4%)		0.100

The proportion of patients with RR ≤ 90 mmHg on arrival is calculated from the patients with available blood pressure values. RR: Blood pressure.

### Analysis of clinical care

The mean length of stay in the trauma room was 62.7 ± 28.4 minutes with no significant difference between the BH sample and the NBH sample (63.9 ± 29.7 versus 62.3 ± 28.0 minutes, p = 0.700). Likewise, the time from arrival at the trauma room to the beginning of the CT scan was similar during BH and NBH with 28.5 ± 18.7 and 27.3 ± 9.5 minutes, respectively, p = 0.637. The proportion of patients, who received a whole-body CT scan was similar with 89.6% (86 patients) and 89.9% (268 patients) in BH and NBH, respectively, p = 0.921 (Table 4).

**Table 4:** **Trauma room management and treatment comparison**

	**n (total)**	**mean (total) ± SD (total)**	**n (BH)**	**mean (BH) ± SD (BH)**	**n (NBH)**	**mean (NBH) ± SD (NBH)**	**p-value**
Time until FAST-Sono (min)	314	6.0 ± 4.5	78	5.7 ± 3.0	236	6.1 ± 4.9	0.763
Time until CT (min)	351	27.6 ± 12.4	86	28.5 ± 18.7	265	27.3 ± 9.5	0.637
Time until discharge from TR (min)	375	62.7 ± 28.4	93	63.9 ± 29.7	282	62.3 ± 28.0	0.700
Time for intubation (min)	22 (5.6%)	4.0 ± 4.0	4 (4.2%)	2.8 ± 2.2	18 (6.0%)	4.2 ± 4.4	0.460 (0.487)
Time for artery access (min)	220 (55.8%)	4.9 ± 3.6	59 (61.5%)	4.8 ± 3.9	161 (54.0%)	4.9 ± 3.4	0.496 (0.202)
Time for CVL (min)	54 (13.7%)	7.2 ± 5.5	15 (15.6%)	6.0 ± 5.6	39 (13.1%)	7.7 ± 5.5	0.084 (0.529)
Time for peripherial access (min)	66 (16.8%)	1.8 ± 1.3	12 (12.5%)	1.8 ± 0.9	54 (18.1%)	1.8 ± 1.4	0.757 (0.200)
Time for thorax-drainage (min)	19 (4.8%)	7.4 ± 4.3	5 (5.2%)	10.4 ± 6.5	14 (4.7%)	6.3 ± 2.7	0.243 (0.542)
Time for splinting (min)	89 (22.6%)	4.1 ± 3.4	23 (24.0%)	4.2 ± 3.9	66 (22.1%)	4.1 ± 3.3	0.658 (0.712)
Time for permanent catheter (min)	229 (58.1%)	2.1 ± 1.4	51 (53.1%)	2.2 ± 1.9	178 (59.7%)	2.0 ± 1.2	0.846 (0.254)

TR: Trauma room, CVL: Central Venous Line.

**Figure 1:**
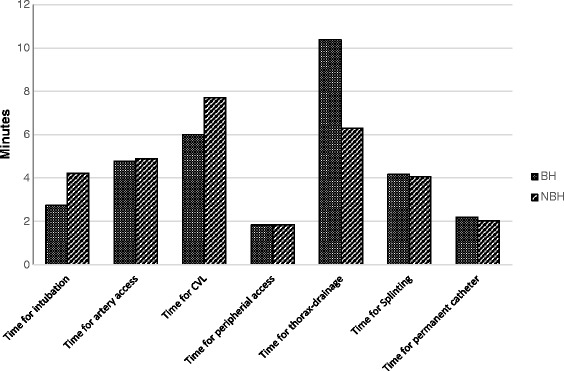
**Comparison of length in time for single interventions.** CVL: Central Venous Line.

In addition no significant difference was observed regarding the time needed to institute arterial lines, intravenous lines, central venous catheter, urinary catheter, and limb splinting (see Table 4 and Figure 1).

### Outcome analysis

**Figure 2:**
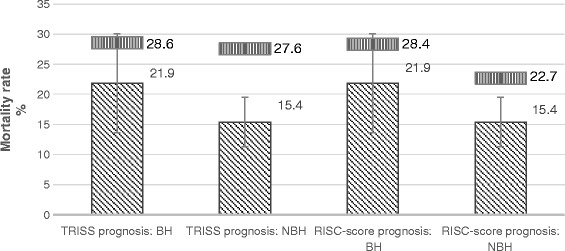
**Observed vs. expected in hospital mortality: A TRISS and RISC-comparison.** The upper bar shows the expected mortality, the lower bar the observed mortality.

The early mortality rate [death within 24 hours: BH: n = 13 (13.5%), NBH: n = 27 (9.1%), p = 0.206], as well as the in-hospital mortality rate [BH: n = 21 (21.9%), NBH: n = 46 (15.4%), p = 0.144] showed trends towards higher mortality rates in the BH-sample. However, these were not statistical significant. The difference between the calculated RISC-prognosis and the observed mortality was 6.4% and 7.2% in the BH and NBH samples, respectively (Figure 2). The TRISS-SMR-Rate was 0.76 (95% CI 0.48–1.05) during BH and 0.56 (95% CI 0.41–0.71) during NBH (see Table 5). On average, patients were intubated for 7.2 ± 10.2 and 7.4 ± 10.0 days (p = 0.856) when admitted in BH and NBH, respectively. The mean length of ICU stay was 11.5 ± 14.9 and 12.0 ± 11.9 days (p = 0.183), and the mean length of hospital stay was 21.8 ± 21.0 and 22.2 ± 16.6 days in BH and NBH (p = 0.268). The mean GOS in both groups was comparable with 3.7 ± 1.6 and 3.9 ± 1.5 in BH and NBH (p = 0.305). The prevalence of MOF (Multi Organ Failure) and sepsis was similar in both groups (Table 6).

**Table 5:** **Comparison of SMR: TRISS and RISC**

	**SMR**	**CI 95%**
BH: TRISS	0.764	0.476-1.053
NBH: TRISS	0.559	0.410-0.707
BH: RISC	0.771	0.480-1.063
NBH: RISC	0.679	0.499-0.860

CI: Confidence interval, SMR: Standardized Mortality Ratio, RISC: Revised Injury Severity Classification Score, TRISS: Trauma and Injury Severity Score.

**Table 6:** **Outcome comparison of BH and NBH**

	**n (total)**	**mean (total) ± SD (total)**	**n (BH)**	**mean (BH) ± SD (BH)**	**n (NBH)**	**mean (NBH) ± SD (NBH)**	**p-value**
GOS	392	3.8 ± 1.5	95	3.7 ± 1.6	297	3.9 ± 1.5	0.305
GOS > = 4	265 (67.6%)		59 (62.1%)		206 (69.4%)		0.188
Died within 24 h (%)	40 (10.2%)		13 (13.5%)		27 (9.1%)		0.206
In hospital mortality (%)	67 (17.0%)		21 (21.9%)		46 (15.4%)		0.144
Days intubated	394	7.4 ± 10.0	96	7.2 ± 10.2	298	7.4 ± 10.0	0.856
Days on ICU	372	11.9 ± 12.7	93	11.5 ± 14.9	279	12.0 ± 11.9	0.183
Days in hospital overall	394	22.1 ± 17.8	96	21.8 ± 21.0	298	22.2 ± 16.6	0.268
Sepsis (%)	38 (9.6%)		10 (10.4%)		28 (9.4%)		0.835
MOF (%)	163 (41.4%)		38 (39.6%)		125 (41.9%)		0.559

GOS: Glasgow Outcome Scale, MOF: Multi Organ Failure.

A sensitivity analysis with different start time (7:00; 7:30; 8:30; 9:00 a.m.) or end time (3:00; 3:30; 4:30; 5:00 p.m.) revealed no major differences in injury severity, treatment or outcome to the chosen interval.

## Discussion

The present study compares the quality of multiple trauma patient care and outcome between BH and NBH. We demonstrated that the time of admission had no measurable impact on a broad spectrum of monitored variables that served as process and quality indicators for trauma care and outcome at the study hospital which is a level-1 trauma center. We therefore conclude that a level-1 trauma center can provide high quality trauma care irrespective of the time of patient admission.

The observed patient sample is comparable to other multiple trauma patient-studies with regard to demographics and grade of injury (ISS: 29.7 ± 13.0/28.8 ± 12.1/median ISS 20 (IQR, 16–26)) [25-27].

Looking at the prehospital parameters, we found no differences between BH and NBH, with the exception of the proportion of air lifted patients, which was significantly lower during NBH. This might be partially attributable to the air rescue-algorithms in the study hospitals catchment area: after sunset, a helicopter can be demanded in addition by a ground emergency physician [28]. It has been shown previously that ordering an air rescue could result in a prolonged prehospital time although this had no impact on the outcome [29]. Interestingly, the rate of prehospital intubations is not affected by the time of admission which underlines the quality of the well-established area-wide 24 h-ground-emergency-service.

Likewise, we found no differences between BH and NBH with regard to the trauma room treatment parameters. It is well accepted that the human work performance decreases at night [30,31], however, an impact of BH and NBH on the monitored care and outcome parameters cannot be demonstrated. Data of the duration of single interventions are scarce. To the best knowledge of the authors, similar studies examining the duration of single interventions around the clock are not available. The lack of external benchmarks limits the comparability of this study. Nevertheless, the different time measurements (time-to-intervention or time period to complete a certain intervention) were comparable in the BH and NBH study sample. This indicates, that the trauma team instituted comparable trauma care around the clock.

Importantly, the core outcome “24 h mortality” was similar in BH and NBH. With respect to comparable patient demographics and injury severity, it could be presumed that the care during NBH was on the same level as during BH. This might be attributable to a broad spectrum of factors, including standardized care algorithms, trauma care training and resources available in the level-1 study hospital. The observed mortality was up to 16% lower than in previous studies though sample characteristics were comparable. This finding is supported by the SMR-calculations of Huber-Wagner et. al. (0.68 – 0.77 vs. 0.85 – 0.98) [25,32]. The clinical outcome of trauma patients in BH versus NBH has been evaluated in very few studies so far [27,32-36]. In general, consistent results were obtained in regard to clinical outcome in BH and NBH, however, the time needed to institute specific interventions was not recorded. In contrast, Di Bartolomeo et al. could demonstrate an off hour effect on trauma patients. This effect was most evident in transferred cases [37]. In other medical emergencies, like stroke [38-41] or myocardial infarction [42,43] previous studies didn’t come to a consistent conclusion regarding the impact of the hospital admission time on the outcome. Conflicting results can be found in the literature concerning time of admittance and outcome [44-48].

We emphasize that it is important to guarantee a constant high level of quality of care for major trauma patients. This includes that structural and trauma team requirements have to be fulfilled. Within our study setting (level-1 trauma center), it seems that there was sufficiently qualified personnel available during business hours as well as during non-business hours [49].

Since this is a single center study, the chosen interval for BH from 8:00 a.m. to 4:00 p.m. represents the shift time of all participating disciplines in particular of the trauma surgery and anaesthesia in the study hospital. In other hospitals, different intervals might fit better.

Despite a large and well-defined patient sample, the present study has a number of limitations. First of all, it is a cohort study with a retrospective design. Second, information on prehospital deaths is not provided since these were not recorded in the database. Third, due to missing data in the trauma registry of the German Trauma Society (TraumaRegister DGU®), TRISS was calculated in 76.6% of all 394 cases only whereas the RISC-score was completed in 98.5%.

Fourth: For various reasons, the research fellow was not permanently present in 105 of 499 cases in the trauma room; therefore these data sets could not be used in this study as is might has biased the results.

Finally, even though the trauma room treatment was standardized, the treating trauma team differed; however, this limitation represents the daily routine in a clinic.

## Conclusions

Based on 394 patients, this is the first study that shows that there were no major differences in characteristics, treatment or outcome between multiple trauma patients admitted during BH and those who were admitted during NBH. This indicates comparable treatment in the sense of quality management regardless of admittance during BH or NBH. A well-trained staff, well-structured organization and other standardizations of the treatment process could be conducive to this result.

## Abbreviations

AIS: Abbreviated Injury Scale
ASA: American Society of Anesthesiologists Classification
ATLS: Advanced Trauma Life Support
BH: Business Hours
CT: Computer Tomography
CVL: Central Venous Line
DGU: German Trauma Society
TR-DGU: TraumaRegister DGU®
EC: Erythrocyte Concentrate
ETC: European Trauma Course
GOS: Glasgow Outcome Scale
ICU: Intensive Care Unit
ISS: Injury Severity Score
MOF: Multi Organ Failure
NBH: Non-Business Hours
NISS: New Injury Severity Score
RISC: Revised Injury Severity Classification Score
SD: Standard Deviation
SMR: Standardized Mortality Ratio
TARN: Trauma Audit & Research Network
TR: Trauma room
TRISS: Trauma and Injury Severity Score
